# Cirrhosis due to hepatic sarcoidosis: A rare presentation

**DOI:** 10.1002/ccr3.8999

**Published:** 2024-06-05

**Authors:** Sumona Islam, Intisar Kamal, Khaled Murshed, Md Abul Kalam Azad

**Affiliations:** ^1^ Department of Gastroenterology Bangabandhu Sheikh Mujib Medical University Dhaka Bangladesh; ^2^ Department of Medicine Bangabandhu Sheikh Mujib Medical University Dhaka Bangladesh

**Keywords:** ACE, cirrhosis, granuloma, sarcoidosis

## Abstract

**Key Clinical Message:**

Sarcoidosis, although predominantly affecting the lungs, can present with cirrhosis, posing diagnostic challenges. Elevated ACE levels and atypical liver enzyme patterns should prompt consideration of sarcoidosis in cryptogenic cirrhosis cases, necessitating comprehensive evaluation including liver biopsy and imaging for accurate diagnosis and timely management.

**Abstract:**

Sarcoidosis is a systemic disease that can affect various organs, leading to a diverse range of clinical manifestations that make diagnosis challenging. Here, we present a case of sarcoidosis in a middle‐aged male who presented with cirrhosis. The cause of cirrhosis remained unknown for 4 years until the development of lymphadenopathy and ground‐glass opacities on lung imaging. A liver biopsy was performed, which revealed noncaseating granulomatous inflammation, thereby identifying sarcoidosis as the cause of cirrhosis. The patient was treated with oral steroids, which slightly improved his liver function over a short period. Given the diverse presentations of sarcoidosis, it should be considered as a possible differential diagnosis in cases of cryptogenic cirrhosis.

## 1 INTRODUCTION

Sarcoidosis is a granulomatous disease of unknown etiology that can affect almost any organ. The prevalence is slightly higher in females and African Americans, with a peak onset age of 20–40 years.^1^, ^2^, ^3^ It most commonly affects the lungs, followed by the lymph nodes.^2^ Liver involvement is not uncommon in sarcoidosis (approximately 11.5%); however, about 80%–85% of cases are asymptomatic.^3^, ^4^ The usual pathology is granuloma formation in the liver, but the incidence of cirrhosis is less than 1%.^3^, ^5^ In this report, we describe the case of a middle‐aged male who presented with a 4‐year history of cirrhosis with no identifiable cause. The patient was subsequently diagnosed with sarcoidosis.

## 2 CASE HISTORY

**FIGURE 1:**
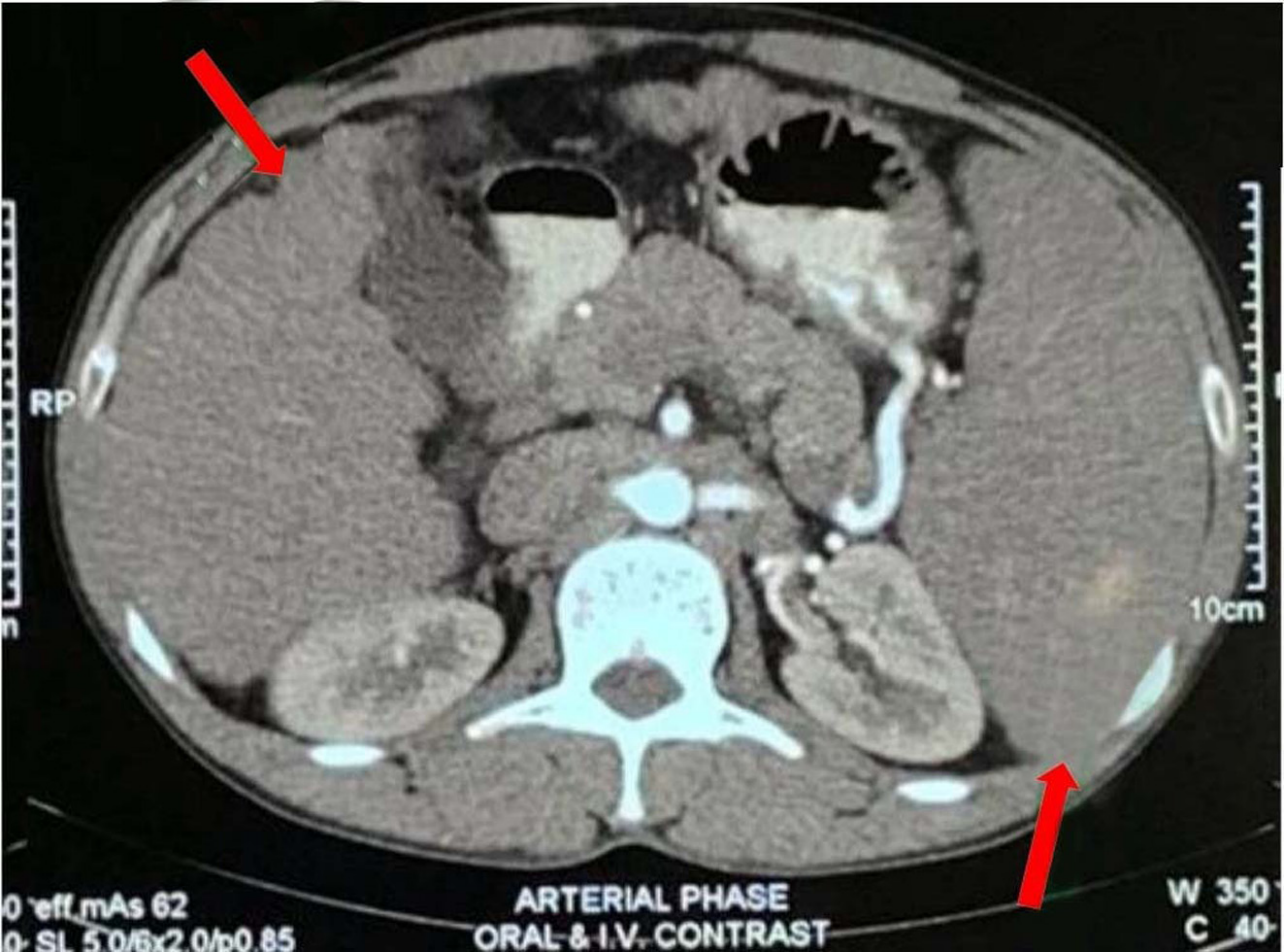
CT scan of abdomen showing nodular liver surface with heterogenous parenchymal attenuation and splenomegaly with nonspecific splenic nodule.

A nonalcoholic, nondiabetic construction worker in his late thirties consulted a physician in 2018 complaining of pruritus. He also experienced significant weight loss (10 kg over 3 months). His liver enzymes were also elevated. Alkaline phosphatase (ALP) and gamma glutamyl transferase (GGT) were markedly raised (487 U/L and 88 U/L, respectively). Aspartate aminotransferase (AST) and alanine aminotransferase (ALT) were slightly increased, but bilirubin was normal. With this cholestatic pattern of hepatic dysfunction, magnetic resonance cholangiopancreatography (MRCP) revealed multiple irregular strictures in the intrahepatic bile ducts. Unfortunately, the patient lost the film and could only provide reports. An abdominal computed tomography (CT) scan revealed liver cirrhosis with nonspecific splenic nodules (Figure 1). Based on these findings, the patient was diagnosed with liver cirrhosis secondary to primary sclerosing cholangitis (PSC). Although liver biopsy was recommended to confirm the diagnosis, the patient refused to undergo the procedure.

Over the course of the next 4 years, he was relatively well, with occasional itching and two episodes of melena. He underwent his first session of esophageal band ligation in 2020 and was administered propranolol (20 mg) twice daily. He did not undergo regular follow‐up.

Approximately 6 months prior, the patient presented with a history of hematemesis and melena for 3 days, along with an increase in the intensity of itching. Urgent endoscopy and a second session of esophageal band ligation were performed. There was no history of intravenous drug abuse, sexual promiscuity, previous surgery, features suggestive of inflammatory bowel disease, or family history of liver disease.

Examination revealed an oriented, cooperative male patient with a body mass index of 24 kg/m^2^. He was mildly anemic, but non‐icteric. There were two enlarged lymph nodes in the jugulodigastric region, both 1 × 1 cm, nontender, soft in consistency, mobile, not fixed to underlying structures, and with no discharging sinus. No clubbing, leukonychia, gynecomastia, or spider angioma were observed. However, the spleen was firm and nontender and markedly enlarged, about 15 cm from the left costal margin along its long axis. The liver was not palpable. Ascites was absent, and both testes were atrophied. Examination of other systems was unremarkable.

## 3 INVESTIGATIONS AND TREATMENT

Investigations revealed that his hemoglobin level was 6.9, that rose to 10.1 after giving two units of blood. AST and ALT levels were more than two times the upper limit of normal. ALP and GGT levels were elevated to 240 U/L and 148 U/L, respectively. Serum albumin and prothrombin time were normal.

**FIGURE 2:**
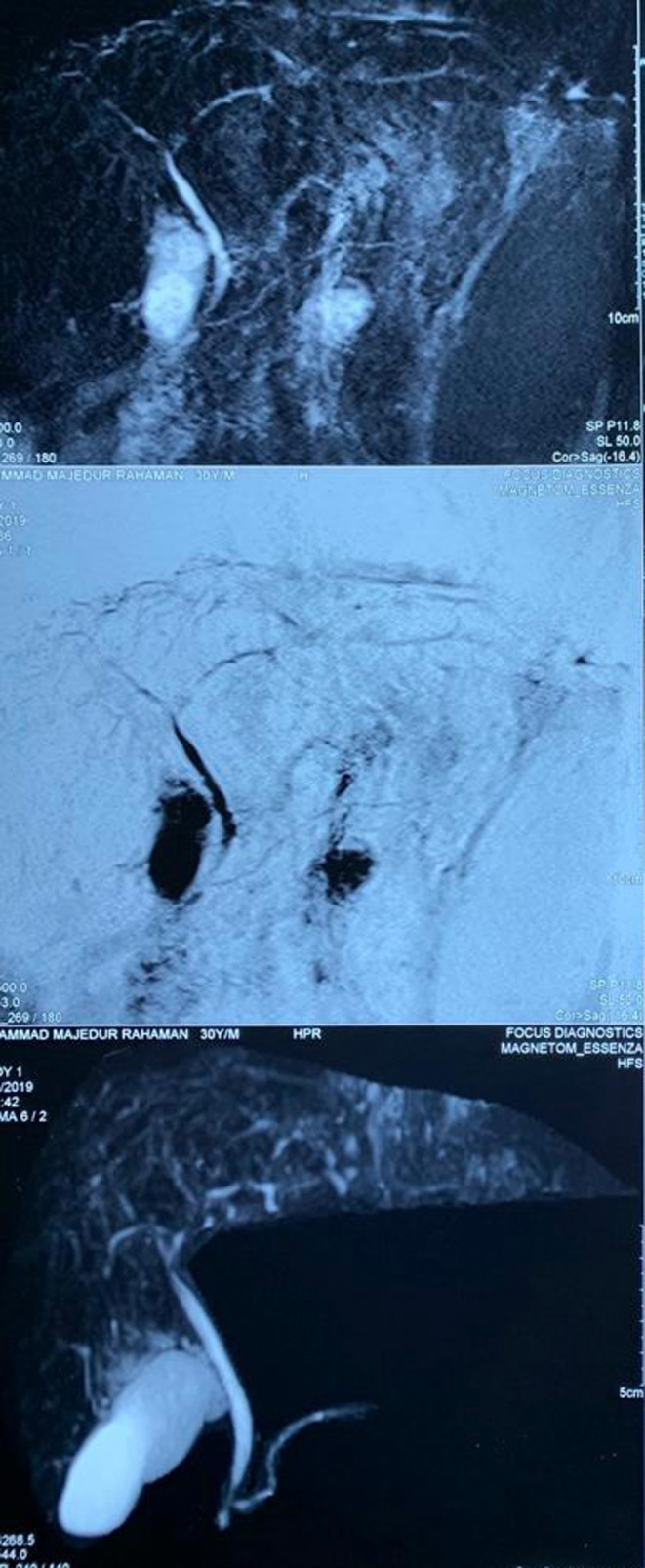
Magnetic resonance cholangiopancreatography.

As the cause of cirrhosis was not previously confirmed, we reassessed the patient. Viral markers for hepatitis B, C, and E and HIV were negative. Serum iron profile, serum ceruloplasmin, and 24‐hour urinary copper levels were normal. Autoantibody screens, including antinuclear antibody, anti‐mitochondrial antibody, smooth muscle antibody, and liver kidney microsomal antibody were all negative. IgG levels were within the normal range and p‐ANCA was negative. Corrected serum calcium levels were normal. Ultrasonography revealed features suggestive of liver cirrhosis, with a suspected space‐occupying lesion, splenomegaly, and mild ascites. Magnetic resonance imaging (MRI) with MRCP showed cirrhosis of the liver with splenomegaly without any intrahepatic bile duct abnormalities (Figure 2).

In light of the patient's cirrhosis accompanied by elevated ALP levels and lymphadenopathy and after ruling out common causes of cirrhosis, we conducted a serum angiotensin‐converting enzyme (ACE) test to explore the possibility of sarcoidosis, which revealed elevated levels.

**FIGURE 3:**
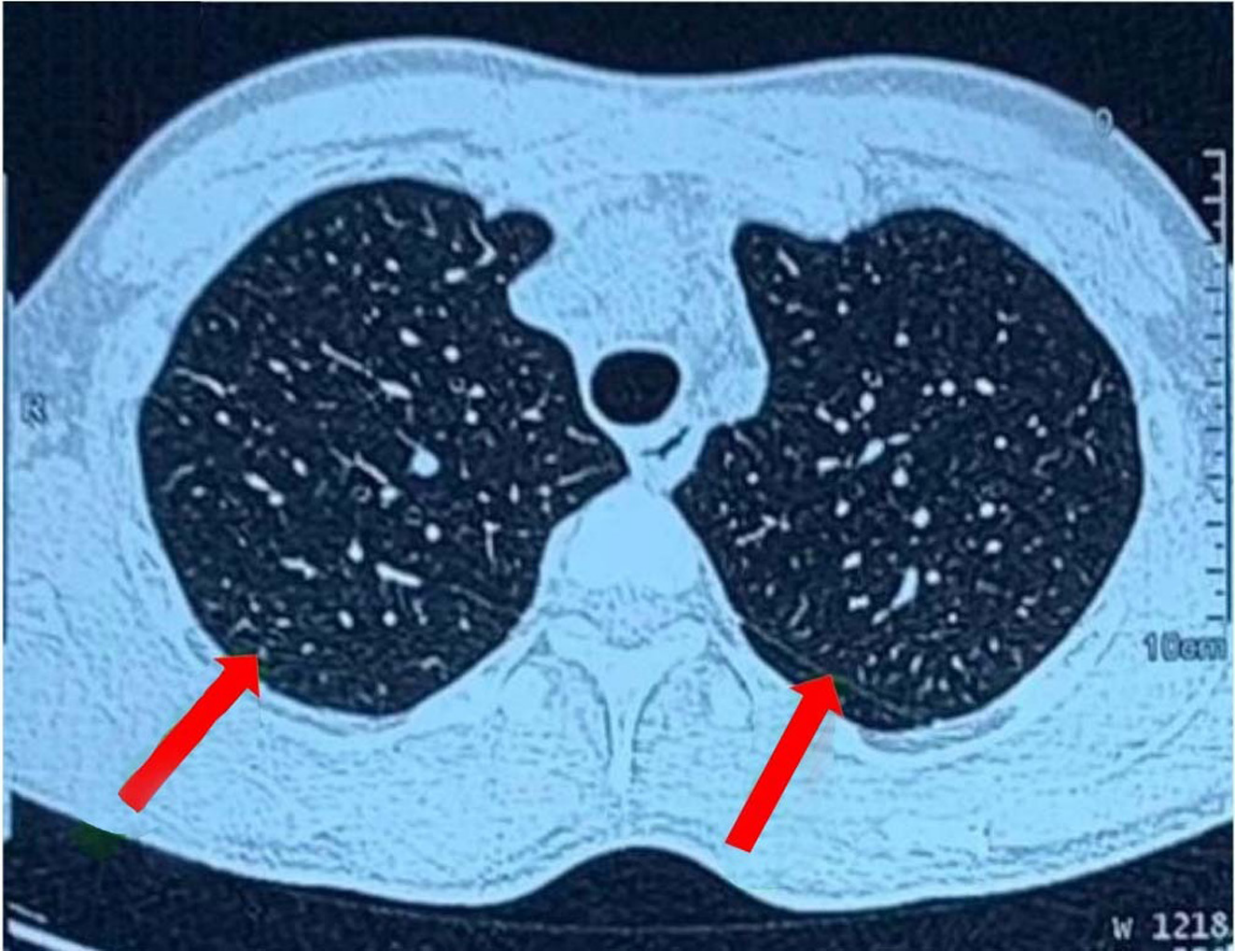
High‐resolution chest computed tomography showing peripheral ground glass opacity.

Despite the absence of cough or respiratory distress, we conducted a high‐resolution chest CT (HRCT) to further investigate the suspicion of sarcoidosis. HRCT revealed multiple areas of peripheral ground‐glass opacities in the right middle and bilateral upper lobes, as well as a few areas of reticular thickening in the bilateral lower lobes. No enlarged mediastinal lymph nodes were observed (Figure 3). Spirometry was normal, but reduced diffusion of carbon monoxide in the lungs was detected, and the 6‐minute walk test showed impaired lung function.

**FIGURE 4:**
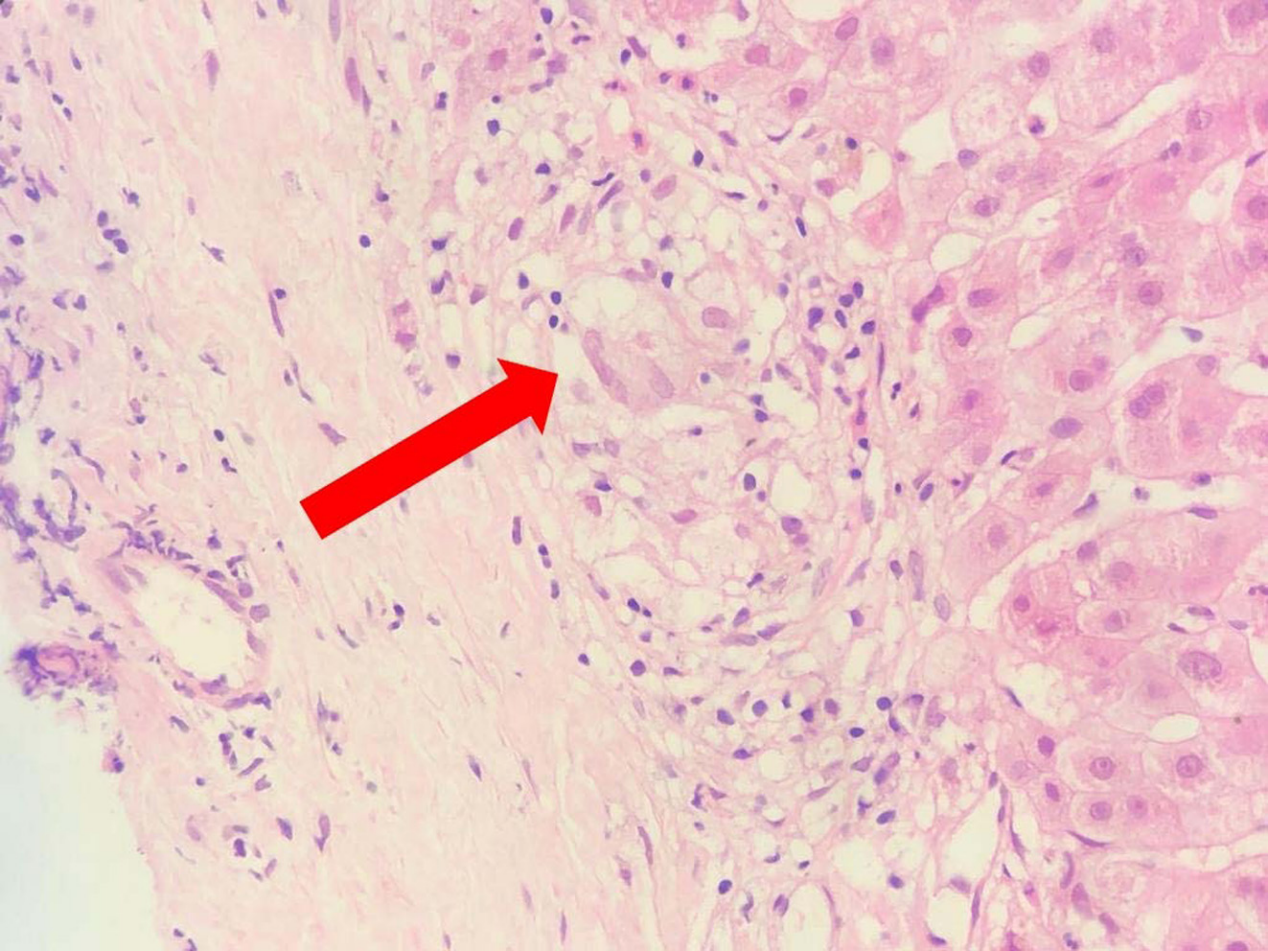
Liver histopathology showing noncaseating granulomatous inflammation.

The patient was counseled for a liver biopsy, and he consented. Histopathological examination revealed noncaseating granulomatous inflammation with fibrotic areas (Figure 4). Staining for acid‐fast bacilli, cytology, and gene experts in the bronchoalveolar lavage yielded negative results. Based on these findings, the patient was diagnosed with decompensated cirrhosis of the liver secondary to sarcoidosis.

The patient was started on prednisolone at 40 mg/day and discharged. The possible side effects of steroids were explained to the patient prior to discharge.

## 4 FOLLOW‐UP

After 3 months of prednisolone treatment, there was a slight improvement in his liver enzymes. However, once cirrhosis develops, the likelihood of liver recovery is low. (Table 1).

**TABLE 1:** Liver function test on admission and 3 months after starting steroid.

LFT	Admission	Follow‐up at 3 months	Reference value
S. bilirubin	22.23 μmol/L	20.52 μmol/L	5.1–20.5 μmol/L
AST	80 U/L	53 U/L	10–34 U/L
ALT	84 U/L	57 U/L	10–49 U/L
ALP	240 U/L	120 U/L	46–116 U/L
GGT	148 U/L	85 U/L	<38 U/L
S. albumin	34 gm/L	35 gm/L	35–50 gm/L
Prothrombin time	12 s	12 s	12 s

## 5 DISCUSSION

Sarcoidosis is a systemic disease characterized by the formation of noncaseating granulomas in various organs. Its precise pathophysiology remains unclear but is believed to result from an exaggerated immune response triggered by an unknown antigen in genetically predisposed individuals.^6^ It can affect multiple organs in the body; however, lung involvement is the most common (90% of cases). Although liver involvement is not uncommon in sarcoidosis, most cases are asymptomatic with only granulomatous inflammation in the liver.^6^ However, asymptomatic liver enzyme elevation occurs in half of hepatic sarcoidosis cases. Symptomatic liver involvement has been observed in approximately 15% of patients. Common symptoms may include abdominal pain and itching.^3^, ^7^

In rare cases, sarcoidosis can lead to cirrhosis. Cirrhosis can occur as a result of extensive granuloma formation, causing fibrosis or secondary biliary cirrhosis due to bile duct inflammation.^6^, ^8^ Although sarcoidosis has been reported to cause cirrhosis in some cases, the initial symptoms are usually related to lung involvement.^9^, ^10^ However, our patient presented with no respiratory symptoms, making the diagnosis of sarcoidosis‐associated cirrhosis difficult. This underscores the importance of considering sarcoidosis as a potential underlying condition in patients presenting with cirrhosis of unknown etiology even in the absence of typical respiratory symptoms.

In sarcoidosis, ALP and GGT levels are typically higher than aminotransferase levels.^3^ Granulomatous bile duct inflammation can mimic PSC. In some reported cases, patients were initially misdiagnosed with PSC.^11^, ^12^ Our patient was initially suspected of having PSC owing to his clinical presentation, but a negative history of inflammatory bowel disease and negative p‐ANCA test results prompted the physician to perform a liver biopsy.

Serum ACE levels were elevated in 75% of the sarcoidosis.^3^ Our patient also showed elevated serum ACE levels. Although other conditions may also cause elevated ACE levels, further investigation is necessary to determine whether sarcoidosis is the underlying cause.

Although there is no established treatment protocol for sarcoidosis‐associated liver involvement, steroids are widely recognized as the primary treatment for cases involving the lungs, eyes, and central nervous system.^13^ Although asymptomatic liver involvement may not require treatment, symptomatic liver disease should be treated. Treatment may lead to the improvement of symptoms and liver function tests in cases of mild to moderate abnormalities. However, improvement is less likely in severe cases of sarcoidosis‐associated liver disease.^13^, ^14^ During the short follow‐up period, our patient demonstrated good improvements in symptoms but biochemical markers were improved slightly. However, cirrhosis is an irreversible condition, and although treatment may slow disease progression, it cannot reverse it.

## AUTHOR CONTRIBUTIONS

1. **Case Involvement and Contribution:**
  - Sumona Islam: Contributes to the diagnosis, treatment and follow‐up of the case.
  - Md Intisar Kamal: Contributes to data collection and interpretation related to the case.
  - Khaled Murshed: Detail involvement in literature review, case background research, or specific medical aspects.
  - Md Abul Kalam Azad: Contributes to the discussion and implications of the case report.
2. **Drafting or Critical Revision:**
  - Sumona Islam: Involves in drafting the case report and significant revisions.
  - Md Intisar Kamal: Contributes to the critical revision and drafting process.
  - Khaled Murshed: Contributes to subsequent critical revisions.
  - Md Abul Kalam Azad: Contributes to critical review stages.
3. **Final Approval:**
  - All authors have reviewed and given final approval for the version of the case report to be submitted for publication.
4. **Accountability and Investigation:**
  - All authors collectively agree to be accountable for the accuracy and integrity of the case report.
  - Commit to addressing any concerns or queries about the case report's content or data.

## FUNDING INFORMATION

No funding.

## CONFLICT OF INTEREST STATEMENT

The authors declare no conflict of interests.

## CONSENT

Written informed consent was obtained from the patient to publish this report in accordance with the journal's patient consent policy.

## Data Availability

All the data related to this case report are available to corresponding author and will be available upon request.
